# Translation and psychometric properties of the King’s Sarcoidosis Questionnaire (KSQ) in German language

**DOI:** 10.1186/s12955-019-1131-z

**Published:** 2019-04-11

**Authors:** Erik Farin, Katja Heyduck, Björn Christian Frye, Surinder S. Birring, Joachim Müller-Quernheim, Jonas Christian Schupp

**Affiliations:** 1 Section of Health Care Research and Rehabilitation Research, Faculty of Medicine, Medical Center- University of Freiburg, Freiburg, Germany; 2 Department of Pneumology, Faculty of Medicine, Medical Center- University of Freiburg, Freiburg, Germany; 3 Division of Asthma, Allergy and Lung Biology, King’s College London, London, UK; 4 Section of Pulmonary, Critical Care and Sleep Medicine, Yale University School of Medicine, New Haven, CT USA

**Keywords:** Sarcoidosis, Questionnaire, German, Quality of life, Human

## Abstract

**Background:**

King’s Sarcoidosis Questionnaire (KSQ) is a novel, validated, health-related quality of life questionnaire on sarcoidosis with 5 scales and 29 items. For future multinational observational and interventional studies on sarcoidosis, a validated German version of the KSQ is needed. The objective of our study is to translate the original KSQ and develop a German version possessing good psychometric properties and with as few modifications as possible.

**Methods:**

We translated the KSQ into German, tested it in structured interviews in sarcoidosis patients, and asked consecutive patients in an outpatient clinic to complete it. We relied on the KSQ’s original version to achieve its psychometric properties in the German version. Structural validity, internal consistency, construct validity, and fit to Rasch model were assessed. Our procedure’s logic meant that in the first step we optimized the item selection in the German version to maximize its psychometric quality. In step two, we assessed the unmodified version‘s properties in comparison to the modified version’s.

**Results:**

One hundred ninety-four patients with sarcoidosis were included and completed the questionnaires. Due to ambiguous factor loadings, four items of the scale “General Health Status” had to be eliminated. Another item was excluded to ensure the Rasch model fit. This modified, 24-item version of the KSQ shows acceptable Rasch model fit and good model fit in confirmatory factor analyses (TLI = 0.90, CFI = 0.91, RMSEA = 0.08). Cronbach’s Alpha ranges from 0.82 to 0.91. Several hypotheses concerning construct validity (e.g., correlations with SF-36) are confirmed or partly confirmed. The measurement properties of the original unmodified version are similar in their construct validity and internal consistency; however, we were unable to confirm structural validity and fit to the Rasch model in the original version.

**Conclusions:**

We translated and validated the German KSQ and report good psychometric properties. The reduced 24-item version has the advantage that all scales are unidimensional and fulfil the requirements of the Rasch model, ensuring its benefits. The original 29-item version, on the other hand, allows us to compare German data to international data however, at the price, of less structural validity and the lack of fit to the Rasch model.

**Trial registration:**

This study was registered in the German Clinical Trials Register (reference number DRKS00010072). Registered January 2016.

**Electronic supplementary material:**

The online version of this article (10.1186/s12955-019-1131-z) contains supplementary material, which is available to authorized users.

## Background

Sarcoidosis is a systemic granulomatous disease most often affecting the lung, the lymph nodes, the eyes, and the skin [1, 2]. Patients with sarcoidosis may suffer from pulmonary symptoms such as cough, dyspnea or chest pain, or systemic symptoms such as fever, weight loss or night sweat. Therefore, sarcoid patients often suffer poorer general health and lower respiratory-specific quality of life [3, 4].

Clinical studies use different endpoints, depending on the main organ involvement studied [5]. However, patient-reported outcomes are routinely measured in clinical trials and often implemented at least as secondary endpoints. The World Association of Sarcoidosis and Other Granulomatous Disorders, therefore, recommends that sarcoidosis studies should include multiple endpoints gauging an ameliorated organ-specific physiology and improved quality of life [5].

King’s Sarcoidosis Questionnaire (KSQ) is a novel, validated, health-related quality of life (HRQOL) questionnaire on sarcoidosis covering five domains (general health status (GHS), medications (M), lung (L), eye (E) and skin (S)) [6]. The KSQ has already been translated into Dutch and validated in a Dutch cohort [7]. It is in use in American (clinical trials identifier: NCT02024555), British (NCT02643732) and Dutch (Netherlands trial register: NTR4328) clinical trials on sarcoidosis. For future multinational observational and interventional studies on sarcoidosis, a validated German version of the KSQ is needed. Hence, we translated the KSQ into German and aimed to validate it in a German cohort of patients with sarcoidosis.

## Methods

### Translation

The English version of the KSQ was used for translation into German and validation in a German cohort [6]. A multistep, forward-backward approach was chosen [8]: First, the English version of the KSQ was independently translated by three authors (EF, JMQ, JCS), merged to a preliminary version, then backward translated by a native speaker. After implementing small suggestions by the original author (SSB), this German pilot version was tested in structured interviews of ten patients with sarcoidosis. Final modifications were implemented and documented based on the structured interviews.

### Subjects

In the outpatient clinic of the department of pneumology, University Hospital Freiburg, consecutive adult, fluent German-speaking patients were screened as to whether they had been diagnosed with sarcoidosis according to the consensus statement of three scientific societies (ERS, ATS, WASOG) [1]. Over a one-year period, 194 patients with sarcoidosis were included in this study after obtaining their informed consent (see Table 1).

**Table 1:** Sample Characteristics

Variable	N_max_ = 194
Age M and SD	52.95 (12.74)
Gender N (%)	
Female	93 (47.9)
Male	100 (51.5)
Nationality N (%)	
German	175 (90.2)
Other	14 (7.2)
Partnership N (%)	
Yes	147 (75.8)
No	38 (19.6)
Level of education N (%)	
Elementary school	56 (28.9)
Secondary school	64 (33.0)
Polytechnic secondary school	2 (1.0)
Technical college qualification	14 (7.2)
University qualification	44 (22.7)
Other or no certificate	7 (3.6)
Employment status N (%)	
Employed	118 (60.8)
Not employed (e.g., retired, housewife or –husband)	59 (30.4)
Duration of illness N (%)	
< 1 year	11 (5.7)
1–2 years	20 (10.3)
3–5 years	60 (30.9)
6–10 years	47 (24.2)
> 10 years	41 (21.1)

Note: Totals not adding up to *N* = 194 are the result of missing values. *N* numbers, *M* mean score, *SD* standard deviation. The percentages refer to the raw values including missing values

### Study procedure

All participants self-completed the following questionnaires and measurements: the KSQ, the Short Form Health Survey (SF-36) [9], the Borg dyspnea scale (CR-10) [10] and the visual analogue scale for dyspnea (VAS-D) [11]. Furthermore, all patients were asked to answer structured questions on their nationality (country of citizenship), sex, duration of the sarcoidosis, sick-days per year, highest educational certificate, employment, and family status.

### Statistical analysis

Our approach is oriented on the original publication of the KSQ (6) and on modern patient-reported outcomes methodology as described in [12]. We have adopted the interpretation of psychometric values of Terwee et al. [13].

#### Distribution properties

To give descriptive information and distribution properties, non-response, item difficulty, skewness and kurtosis were computed (Additional file 1: Table S1). Items with ceiling or floor effects (exceeding 50% of values in the extreme categories) were determined. On the scale-value level, we considered more than 15% of values in the extreme categories as an indicator for ceiling or floor effects.

#### Exploratory factor analysis (EFA)

We conducted an EFA (software: IBM SPSS version 24, principal component analysis with oblique rotation). To determine the number of factors to be extracted, the following criteria were used: (a) factor loading (an item was removed if it did not load unambiguously on the extracted factors, factor loading > = 0.50 on exactly one factor; factor loading < 0.40 on all other factors), and (b) congruence with the original KSQ.

#### Item response theory (IRT) analyses

We used the 1-parameter IRT model (Rasch model) for item response theory analyses (software: WINSTEPS version 3.68), as it is an effective tool for clinical applications [14]. It also provides stable parameter estimates for even small case numbers [15]. Every KSQ scale was analyzed separately. Infit and outfit mean square statistics (Infit MNSQ, Outfit MNSQ) were used as goodness-of-fit statistics. Poor item fit was defined as an infit or outfit < 0.6 or > 1.4 [16]. Items with a poor infit or outfit were eliminated. The Person Separation Index (PSEP) was computed to assess the KSQ’s ability to discriminate patients with different levels of impairments. The PSEP was used as an indicator of the fit statistics’ reliability. Reliability of 0.8 is achieved for PSEP > 2, and reliability of 0.90 for PSEP > 3.

#### Confirmatory factor analysis

Based on the EFA factor solution and results of the Rasch model, we conducted a confirmatory factor analyses (CFA) using IBM SPSS AMOS 24 software. A five-factor model (corresponding with the KSQ’s five scales) was tested. Model fit was evaluated using the Comparative Fit Index (CFI, [17]), Tucker–Lewis index (TLI, [18]), root mean square error of approximation (RMSEA) and standardized root mean square residual (SRMR). CFI and TLI values > 0.90 are an indication of good fit. RMSEA values < 0.10 suggest a moderate fit; values < 0.05 a good fit [17]. The SRMR value should be under 0.08 [17]. Satisfactory model fit is assumed whenever at least three out of four parameters produce good values [19]. If model fit was low, a bifactor model was computed to test if a model with a general factor and five group factors (corresponding to the KSQ scales) would better account for our data.

#### Internal consistency

Cronbach’s Alpha was calculated. Values between 0.75 and 0.95 are considered good. The item-total correlation should be between 0.20 and 0.80.

#### Construct validity

To evaluate construct validity, we tested the hypotheses below.

1. As the Borg scale and VAS Dyspnea assess lung symptoms, we expect these parameters to correlate closely (r > =0.60) with the KSQ Lung scale, and we expect low or moderate correlations (0.20–0.60) with the other KSQ scales.
2. We expect high correlations (r > =0.60) between the KSQ General Health scale and the SF-36 Physical and Mental Component scales, as those scales measure two facets of general health status. The correlations between the SF-36 Physical and Mental Component scales and other KSQ scales are assumed to be low or moderate.

## Results

### Structured interviews

Ten patients with sarcoidosis answered the pilot German KSQ in structured interviews. The sarcoid patients had no problems understanding or answering the questionnaire. To improve comprehensibility, minor modifications were made in the final German KSQ. The final version is presented in Additional file 1: Table S1.

### Step 1: development of an optimized German version allowing item selection

#### Distribution properties

Eight items show floor effects (1 item on the General Health Status Scale, 2 items on the Lung, Medication and Skin scale, respectively, and 1 item on the Eyes scale). The 3 medication items reveal a high non-response rate up to 11.3%. The latter result presumably reflects the fact that some patients take no medicine. On the scale-value level, we observed floor effects in conjunction with the Medication, Skin, and Eyes scales. 161 of 194 patients (83.0%) answered all items. If the Medication Scale is disregarded, 176 persons filled in all questions (90.7%).

#### Exploratory factor analysis

The Lung, Medication, Skin, and Eyes scales were reproduced exactly as in the original KSQ (Additional file 2: Table S2). As four items on the “General Health Status” scale (items 6 to 9) exhibited ambiguous factor loadings, we decided to eliminate them.

#### Item response theory (IRT) analyses

To ensure fit to the Rasch model, items were recoded using a five-point Likert scale. Response categories 2 and 3 (most of the time or a good bit of the time) as well as 5 and 6 (a little of the time vs. hardly any of the time) were merged. Item 11 (“My cough has caused me pain/discomfort”) was eliminated, as the infit and outfit values exceeded the acceptable cut-offs.

After making those changes, all the scales’ fit values fell in the adequate range (General Health Status Scale GHS 0.71–1.37, Lung Scale 0.80–1.35, Medication Scale 0.74–1.35, Skin Scale 0.73–1.17, Eyes Scale 0.80–1.27). The PSEP reveals good values for the scales GHS (2.96), Lung (2.34), and Eyes (2.38). For the scales with only 3 items (Medication and Skin), the values are below 2.

#### Confirmatory factor analysis

Table 2 presents the CFA results. The original CFA model (without items 6, 7, 8, 9, 11) demonstrated an unsatisfactory fit. The bifactor model does not lead to a better fit. As modification indices suggested that model fit would improve provided correlated error terms were included, we added three theoretically plausible correlated error terms. The first three items on the Eyes scale (dry eyes, red eyes, difficulty with bright lights) exhibit similar content and seem to possess a joint component not entirely explained by the underlying factor. Furthermore, the error terms in the Lung scale’s two items were correlated (My chest has felt tight, I have had episodes of breathlessness). Here, too, the items’ contents resemble each other more than those in the total scale, which addresses different symptoms such as coughing. The modified CFA model with correlated error terms displays a good model fit (Table 2).

**Table 2:** Global Fit Indices of Confirmatory Factor Analyses (24 item version)

	TLI	CFI	RMSEA	RMSEA 90% CI	SRMR
Only scale factors: original model	0.86	0.87	0.10	0.09–0.10	0.07
Bifactor model (unmodified)	0.86	0.89	0.09	0.08–0.10	0.14
Only scale factors: Modified model	0.90	0.91	0.08	0.07–0.09	0.07

Abbreviations: *TLI* Tucker–Lewis index, *CFI* comparative fit index, *RMSEA* root mean square error of approximation, *SRMR* standardized root mean square residual

#### Internal consistency

Table 3 presents internal consistency values based on the KSQ German version with 24 items. Cronbach’s Alpha values are good; the item-total correlation falls within the acceptable range for all scales.

**Table 3:** Internal Consistency

Scale and items	N	Items	Item-Total correlation	Mean Inter-Item correlation	Cronbach’s alpha
General Health Status Includes items 01, 02, 03, 04, 05 and 10	186	6	0.59–0.81	0.60	0.90
Lung Includes items 12, 13, 14, 15 and 16	189	5	0.65–0.78	0.64	0.90
Medication Includes items 17, 18 and 19	172	3	0.58–0.77	0.62	0.82
Skin Includes items 20, 21 and 22	192	3	0.67–0.73	0.63	0.83
Eyes Includes items 23, 24, 25, 26, 27, 28 and 29	187	7	0.68–0.79	0.59	0.91

#### Construct validity

Hypothesis 1 is confirmed (see Table 4). The correlation between the KSQ Lung scale and Borg Scale and VAS Dyspnea is −.64 and − .67. The other KSQ scales’ correlations with the Borg Scale and VAS Dyspnea are in the −.23 to −.44 range.

**Table 4:** Concurrent Validity

	Kings Sarcoidosis Questionnaire				
	General Health Status	Lung	Medication	Skin	Eyes
SF-36 Physical Component Score	.47	.67	.28	.30	.37
SF-36 Mental Component Score	**.63**	.27	.32	.26*	.21*
Borg scale	**−.44**	**−.64**	**−.39**	**−.30**	**−.38**
VAS Dyspnoea	**−.35**	**−.67**	**−.33**	−.23*	**−.27**

Notes: For construct validity analysis, estimated person measures for KSQ scales as calculated in Rasch-analysis were used. Pearson’s correlations, all *p* < 0.001 except * *p* < .01. Interpretation guide: KSQ scales: high values = low burden, SF-36 scales: high values = high manifestation of related dimensions’ content, Borg scale: high values = high dyspnoea, VAS Dyspnoea: high values = high dyspnoea. Correlations that confirm the hypothesized relationships are marked in bold

Hypothesis 2 is only partly confirmed. The correlation between the KSQ General Health scale and SF-36 Mental Component is high (r = .63), as expected, but the correlation with the Physical Component is only moderate (r = .47). The correlations between the SF-36 Physical and Mental Component scales and other KSQ scales are low to moderate with the exception of the association between the SF-36 Physical Component and KSQ Lung scale (r = .67).

### Step 2: psychometric properties of the unmodified German version

To compare the properties of our optimized 24-item version with those of the original version (and thereby expose advantages and disadvantages), we illustrate the psychometric properties of the 29-item German version in a shortened format. Additional file 3: Table S3 and Additional file 4: Table S4 present Cronbach’s Alpha values and construct validity hypothesis testing with this version. The General Health Status Scale’s mean inter-item correlation is lower in the original version, but Cronbach’s Alpha is nearly the same for all scales. As with the Rasch-scaled version, construct validity hypothesis 1 is confirmed and hypothesis 2 is only partly confirmed (the association between the SF-36 Physical Component and KSQ Lung scale is inappropriately high, r = .70). But with the original version, the correlation between KSQ General Health scale and SF-36 Physical Component falls within the expected range (r = 0.60). Confirmatory factor analysis reveals an unsatisfactory fit (TLI = 0.82, CFI = 0.84, RMSEA = 0.09, SRMR = 0.07). Our Rasch analyses showed that the infit and outfit values of 5 Items fell outside the acceptable range.

## Discussion

The psychometric properties of the German KSQ (modified version with 24 items) are good. We provide evidence of reliability, Rasch model fit, and internal consistency as well as structural and construct validity. Concerning the Lung, Skin, Eye and Medication scales, there is perfect correspondence between the original English and German versions. However, the KSQ’s General Health Status scale could not be replicated. In the EFA, the factor loadings of four items on that scale are below 0.30. A confirmatory test of the scale’s unidimensionality with structural equation modeling (data not shown in the results section) demonstrates a poor model fit (with all fit indices exceeding the acceptable range). The items omitted assess worries and embarrassment as well as pain; the remaining items primarily assess fatigue as well as anxiety and depressive symptoms. Considering the general health status of sarcoidosis patients as a unidimensional concept appears to be difficult. This is not surprising, as these patients’ impairments and complaints are heterogeneous and complex. Interestingly, neither the KSQ’s original publication (6), nor studies addressing KSQ translations in other languages (7), have reported data on unidimensionality and structural validity.

Considering that the German version of the KSQ General Health Status scale focuses on fatigue, anxiety, and depression, its use is justified. But if one intends to capture a broad perspective of health status impairments in sarcoidosis patients, it may be advisable to use other well-established instruments that assess patients’ psycho-social problems such as worries, coping deficits, and pain.

Disregarding the problems we encountered with the general health status scale, the German KSQ’s psychometric properties (internal consistency, fit to the Rasch model, indication of construct validity) resemble the original version’s. However, to ensure Rasch model fit, we had to merge two response categories, reducing the 7-point Likert scale to a 5-point scale. It remains unclear whether this is due to the limited ability of patients to distinguish different levels of symptom severity or to a translation artefact (reflecting the difficulties finding conceptual equivalents to the English response choices (see the Discussion in [20]).

As some users may emphasize comparability with international data, we analyzed the original German KSQ’s properties with 29 items and the 7-point Likert scale. The measurement properties of the original unmodified version are similar in their construct validity and internal consistency; however, we were unable to confirm structural validity and fit to the Rasch model. Thus the 24-item version’s advantages over the original 29-item version result from their factorial validity and fit to the Rasch model.

The strengths of this study are the use of modern patient-reported outcomes methodology, including item response modeling and confirmatory factor analyses. But there are some limitations; we have no data on test-retest-reliability, and we did not validate our modified German KSQ in an independent cohort. Further validity testing employing physiological parameters of pulmonary function and proving associations between KSQ scales and apparatus measures is pending. Our sample size suffices for our statistical analyses, but referring to rules of thumb concerning sample sizes in exploratory factor analyses (e.g. the rule of 10: there should be at least 10 persons for each item in the instrument being used [21]); several more individuals would have been favorable. The majority of our analyses include only persons with values on all scales, so sarcoidosis patients without medication (about 10%) were mostly omitted. Furthermore, 76.2% of the patients had an illness lasting 3 years or more, thus most of our patients suffer from chronic sarcoidosis, leading to less explanatory power from patients with acute sarcoidosis. Future analyses of the KSQ’s German version should address differential item functioning (DIF), i.e., DIF by language (English versus German).

## Conclusion

Taken together, we were able to translate and validate the German KSQ and report good psychometric properties in a reduced 24-item version. This modified version has the advantage that all scales are unidimensional and fulfill the Rasch model’s requirements, ensuring its benefits such as sample invariance, convenient handling of missing values, and good clinical interpretability, as person and item parameters are scaled within the same dimension. The original 29-item version, on the other hand, enables us to compare German data with international data, as the KSQ has been translated into over 10 languages. The German KSQ’s potential applications are manifold. First, it could be used to monitor sarcoidosis patients during routine check-ups, as it is quick to fill out. Second, the German KSQ (29-item version) enables German cohorts to participate in multinational clinical sarcoidosis trials employing KSQ. Third, the German KSQ provides clinical researchers with a tool to study the influence of objectifiable sarcoidosis disease activity and organ impairment on the disease’s perception and symptoms.

## Additional files

Additional file 1: Items Analysis: German version of the Kings Sarcoidosis Questionnaire. (DOCX 51 kb)

Additional file 2: Exploratory Factor Analysis: German version of the Kings Sarcoidosis Questionnaire. (DOCX 46 kb)

Additional file 3: Internal Consistency of the original version (29 items, seven-point Likert scale). (DOCX 35 kb)

Additional file 4: Concurrent Validity of the original version (29 items, seven-point Likert scale). (DOCX 35 kb)

## Abbreviations

CFA: Confirmatory factor analysis
CFI: Comparative Fit Index
EFA: Exploratory factor analysis
KSQ: King’s Sarcoidosis Questionnaire
RMSEA: Root mean square error of approximation
SRMR: Standardized root mean square residual
TLI: Tucker–Lewis index
