# Supplementary material for: Translation and psychometric properties of the King’s Sarcoidosis Questionnaire (KSQ) in German language
Source: Health Qual Life Outcomes. 2019 Apr 11;17:62. doi: 10.1186/s12955-019-1131-z (PMC6460543; doi:10.1186/s12955-019-1131-z)
Supplement: Supplementary file 1 — Items Analysis: German version of the Kings Sarcoidosis Questionnaire. (DOCX 51 kb) [file 12955_2019_1131_MOESM1_ESM.docx]

**Supplement Table 1.** **Items Analysis: German version of the Kings Sarcoidosis Questionnaire**

| **Items**  In the last 2 weeks…  *In den letzten 2 Wochen…* | | **Non-response** | | **Mean values** | | **Item difficulty** | **Skew-ness** | **Kurtosis** | **Distribution of extreme values** | |
| --- | --- | --- | --- | --- | --- | --- | --- | --- | --- | --- |
|  |  | N | % | M | SD | d | S | K | %  undermost extreme  (7 = never / none at all) | %  upmost extreme  (1 = always / a huge amount) |
| 1 | I have felt frustrated  *fühlte ich mich entmutigt* | 2 | 1.0 | 5.17 | 1.63 | 0.60 | -0.47 | -0.96 | 28.6 | 0.5 |
|  |  |  |  |  |  |  |  |  |  |  |
| 2 | I have had trouble concentrating  *hatte ich Schwierigkeiten mich zu konzentrieren* | 0 | 0.0 | 4.99 | 1.58 | 0.56 | -0.25 | -1.03 | 23.7 | 0.5 |
|  |  |  |  |  |  |  |  |  |  |  |
| 3 | I have lacked motivation  *mangelte es mir an Antrieb* | 1 | 0.5 | 4.41 | 1.63 | 0.45 | -0.04 | -0.80 | 13.0 | 3.6 |
|  |  |  |  |  |  |  |  |  |  |  |
| 4 | I have felt tired  *fühlte ich mich müde* | 3 | 1.5 | 3.64 | 1.67 | 0.33 | 0.41 | -0.52 | 7.9 | 9.4 |
|  |  |  |  |  |  |  |  |  |  |  |
| 5 | I have felt anxious  *fühlte ich mich ängstlich* | 3 | 1.5 | 5.52 | 1.51 | 0.67 | -0.68 | -0.69 | 38.2 | 0.0 |
|  |  |  |  |  |  |  |  |  |  |  |
| 6 | I have felt aches and pains in my muscles/joints  *hatte ich Schmerzen in meinen Muskeln / Gelenken* | 0 | 0.0 | 4.14 | 1.98 | 0.43 | 0.08 | -1.19 | 19.6 | 9.8 |
|  |  |  |  |  |  |  |  |  |  |  |
| 7 | I have felt embarrassed  *hatte ich mich geschämt* | 1 | 0.5 | 6.44 | 1.19 | 0.89 | -2.32 | 5.16 | 76.7 | 1.0 |
|  |  |  |  |  |  |  |  |  |  |  |
| 8 | I have worried about my weight  *machte ich mir Sorgen über mein Gewicht* | 0 | 0.0 | 4.73 | 2.07 | 0.54 | -0.40 | -1.11 | 32.0 | 11.3 |
|  |  |  |  |  |  |  |  |  |  |  |
| 9 | I have worried about my sarcoidosis  *hatte ich Sorgen wegen meiner Sarkoidose* | 1 | 0.5 | 4.25 | 1.83 | 0.44 | -0.18 | -0.87 | 13.5 | 10.9 |
|  |  |  |  |  |  |  |  |  |  |  |
| 10 | Tiredness has interfered with my normal social activities  *hat Müdigkeit meine normalen sozialen Aktivitäten behindert* | 1 | 0.5 | 4.73 | 1.99 | 0.54 | -0.24 | -1.38 | 30.6 | 3.6 |
|  |  |  |  |  |  |  |  |  |  |  |
| 11 | My cough has caused me pain/discomfort  *hat mein Husten Schmerzen/Unbehagen verursacht* | 4 | 2.1 | 5.92 | 1.53 | 0.76 | -1.39 | 0.99 | 54.2 | 1.6 |
|  |  |  |  |  |  |  |  |  |  |  |
| 12 | I have been breathless climbing stairs or walking up slight inclines  *hatte ich Atemnot beim Treppensteigen oder leichten Bergaufgehen* | 3 | 1.5 | 4.48 | 2.04 | 0.49 | -0.20 | -1.20 | 26.2 | 11.0 |
|  |  |  |  |  |  |  |  |  |  |  |
| 13 | I have had to take deep breaths, also known as “air hunger”  *musste ich tiefe Atemzüge nehmen, auch Lufthunger genannt* | 2 | 1.0 | 5.01 | 1.85 | 0.58 | -0.49 | -0.91 | 32.3 | 4.7 |
|  |  |  |  |  |  |  |  |  |  |  |
| 14 | My chest has felt tight  *spürte ich Enge in der Brust* | 2 | 1.0 | 5.46 | 1.66 | 0.66 | -0.77 | -0.42 | 42.2 | 1.6 |
|  |  |  |  |  |  |  |  |  |  |  |
| 15 | I have had episodes of breathlessness  *kam es zu Atemnot* | 3 | 1.5 | 5.68 | 1.68 | 0.72 | -1.08 | 0.09 | 50.8 | 2.6 |
|  |  |  |  |  |  |  |  |  |  |  |
| 16 | I have experienced chest pains  *erlebte ich Brustschmerzen* | 2 | 1.0 | 5.80 | 1.54 | 0.73 | -1.27 | 1.00 | 50.0 | 2.6 |
|  |  |  |  |  |  |  |  |  |  |  |
| 17 | I have worried about side effects of my medication for sarcoidosis  *habe ich mir Sorgen über Nebenwirk. meiner Sarkoidose-Med. gemacht* | 19 | 9.8 | 5.06 | 2.05 | 0.61 | -0.63 | -0.99 | 40.6 | 7.4 |
|  |  |  |  |  |  |  |  |  |  |  |
| 18 | I have felt worse because of my medication for sarcoidosis  *habe ich mich wegen meiner Sarkoidose-Medikamente schlecht gefühlt* | 21 | 10.8 | 5.75 | 1.85 | 0.74 | -1.26 | 0.27 | 59.5 | 4.0 |
|  |  |  |  |  |  |  |  |  |  |  |
| 19 | I have gained weight because of my medication for sarcoidosis  *habe ich wegen meiner Sarkoidose-Medikamente zugenommen* | 22 | 11.3 | 5.41 | 2.17 | 0.69 | -0.97 | -0.64 | 58.1 | 9.3 |
|  |  |  |  |  |  |  |  |  |  |  |
| 20 | I have been bothered by my skin problems  *haben mich Hautprobleme gestört* | 1 | 0.5 | 5.36 | 1.96 | 0.66 | -0.84 | -0.67 | 48.2 | 4.7 |
|  |  |  |  |  |  |  |  |  |  |  |
| 21 | I have been concerned about changes in colour of my skin lesions  *habe ich mir Sorgen wegen Farbänderungen meines Hautbefalls gemacht* | 2 | 1.0 | 6.07 | 1.58 | 0.80 | -1.73 | 1.91 | 64.6 | 2.1 |
|  |  |  |  |  |  |  |  |  |  |  |
| 22 | I have been embarrassed about my skin  *habe ich mich wegen meiner Haut geschämt* | 0 | 0.0 | 6.40 | 1.35 | 0.87 | -2.46 | 5.41 | 77.8 | 1.5 |
|  |  |  |  |  |  |  |  |  |  |  |
| 23 | I have had dry eyes  *hatte ich trockene Augen* | 2 | 1.0 | 5.01 | 2.09 | 0.60 | -0.57 | -1.10 | 40.6 | 7.8 |
|  |  |  |  |  |  |  |  |  |  |  |
| 24 | I have had difficulty with bright lights  *hatte ich Schwierigkeiten bei hellem Licht* | 1 | 0.5 | 4.94 | 2.02 | 0.58 | -0.45 | -1.15 | 38.3 | 6.7 |
|  |  |  |  |  |  |  |  |  |  |  |
| 25 | My eyes have been red  *waren meine Augen gerötet* | 1 | 0.5 | 5.47 | 1.74 | 0.67 | -0.87 | -0.27 | 44.0 | 3.6 |
|  |  |  |  |  |  |  |  |  |  |  |
| 26 | I have had pain in/or around the eyes  *hatte ich Schmerzen in den oder um die Augen* | 1 | 0.5 | 5.77 | 1.65 | 0.73 | -1.18 | 0.40 | 53.9 | 2.6 |
|  |  |  |  |  |  |  |  |  |  |  |
| 27 | I have had difficulty reading  *hatte ich Schwierigkeiten beim Lesen* | 0 | 0.0 | 5.04 | 2.00 | 0.60 | -0.51 | -1.06 | 40.7 | 6.2 |
|  |  |  |  |  |  |  |  |  |  |  |
| 28 | I have had blurred vision  *habe ich verschwommen gesehen* | 1 | 0.5 | 5.30 | 1.84 | 0.64 | -0.63 | -0.91 | 43.5 | 2.6 |
|  |  |  |  |  |  |  |  |  |  |  |
| 29 | I have been worried about my eyesight  *habe ich mir Sorgen wegen meiner Sehkraft gemacht* | 1 | 0.5 | 5.15 | 2.04 | 0.63 | -0.63 | -1.07 | 44.6 | 4.7 |

Notes: N = 194, M = mean value with higher scores indicating lower agreement to the item, SD = standard deviation, d = item difficulty (high: > 0.80, medium: 0.80-0.20, low: < 0.20), s = skewness, k = kurtosis.
