# Supplementary material for: Translation and psychometric properties of the King’s Sarcoidosis Questionnaire (KSQ) in German language
Source: Health Qual Life Outcomes. 2019 Apr 11;17:62. doi: 10.1186/s12955-019-1131-z (PMC6460543; doi:10.1186/s12955-019-1131-z)
Supplement: Supplementary file 2 — Exploratory Factor Analysis: German version of the Kings Sarcoidosis Questionnaire. (DOCX 46 kb) [file 12955_2019_1131_MOESM2_ESM.docx]

**Supplement table 2.**

**Exploratory Factor Analysis: German version of the Kings Sarcoidosis Questionnaire**

|  | **Factor** | **1** | **2** | **3** | **4** | **5** |
| --- | --- | --- | --- | --- | --- | --- |
|  | **Eigenvalue** | 10.92 | 3.30 | 2.07 | 1.67 | 1.57 |
|  | **% of variance** | 37.67 | 11.39 | 7.14 | 5.82 | 5.40 |
| 1 | I have felt frustrated  *fühlte ich mich entmutigt* |  | .745 |  |  |  |
|  |  |  |  |  |  |  |
| 2 | I have had trouble concentrating  *hatte ich Schwierigkeiten mich zu konzentrieren* |  | .951 |  |  |  |
|  |  |  |  |  |  |  |
| 3 | I have lacked motivation  *mangelte es mir an Antrieb* |  | .961 |  | -.204 |  |
|  |  |  |  |  |  |  |
| 4 | I have felt tired  *fühlte ich mich müde* |  | .848 |  |  |  |
|  |  |  |  |  |  |  |
| 5 | I have felt anxious  *fühlte ich mich ängstlich* |  | .730 |  |  |  |
|  |  |  |  |  |  |  |
| 6 | I have felt aches and pains in my muscles/joints  *hatte ich Schmerzen in meinen Muskeln / Gelenken* | .219 | .260 |  |  |  |
|  |  |  |  |  |  |  |
| 7 | I have felt embarrassed  *hatte ich mich geschämt* |  | .240 |  | .249 | .308 |
|  |  |  |  |  |  |  |
| 8 | I have worried about my weight  *machte ich mir Sorgen über mein Gewicht* |  |  |  | .366 |  |
|  |  |  |  |  |  |  |
| 9 | I have worried about my sarcoidosis  *hatte ich Sorgen wegen meiner Sarkoidose* |  | .227 | .310 | .474 |  |
|  |  |  |  |  |  |  |
| 10 | Tiredness has interfered with my normal social activities  *hat Müdigkeit meine normalen sozialen Aktivitäten behindert* |  | .779 |  |  |  |
|  |  |  |  |  |  |  |
| 11 | My cough has caused me pain/discomfort  *hat mein Husten Schmerzen/Unbehagen verursacht* |  |  | .606 |  |  |
|  |  |  |  |  |  |  |
| 12 | I have been breathless climbing stairs or walking up slight inclines  *hatte ich Atemnot beim Treppensteigen oder leichten Bergaufgehen* |  |  | .916 |  |  |
|  |  |  |  |  |  |  |
| 13 | I have had to take deep breaths, also known as “air hunger”  *musste ich tiefe Atemzüge nehmen, auch Lufthunger genannt* |  |  | .927 |  |  |
|  |  |  |  |  |  |  |
| 14 | My chest has felt tight  *spürte ich Enge in der Brust* |  |  | .805 |  |  |
|  |  |  |  |  |  |  |
| 15 | I have had episodes of breathlessness  *kam es zu Atemnot* |  |  | .891 |  |  |
|  |  |  |  |  |  |  |
| 16 | I have experienced chest pains  *erlebte ich Brustschmerzen* |  |  | .626 |  |  |
|  |  |  |  |  |  |  |
| 17 | I have worried about side effects of my medication for sarcoidosis  *habe ich mir Sorgen über Nebenwirk. m. Sarkoidose-Med. gemacht* |  |  |  | .846 |  |
|  |  |  |  |  |  |  |
| 18 | I have felt worse because of my medication for sarcoidosis  *habe ich mich wegen meiner Sarkoidose-Med. schlecht gefühlt* |  |  |  | .714 |  |
|  |  |  |  |  |  |  |
| 19 | I have gained weight because of my medication for sarcoidosis  *habe ich wegen meiner Sarkoidose-Medikamente zugenommen* |  | -.337 |  | .908 |  |
|  |  |  |  |  |  |  |
| 20 | I have been bothered by my skin problems  *haben mich Hautprobleme gestört* |  |  |  |  | .906 |
|  |  |  |  |  |  |  |
| 21 | I have been concerned about changes in colour of my skin lesions  *habe ich mir Sorgen wegen Farbänderungen m. Hautbefalls gemacht* |  |  |  |  | .874 |
|  |  |  |  |  |  |  |
| 22 | I have been embarrassed about my skin  *habe ich mich wegen meiner Haut geschämt* |  |  |  |  | .863 |
|  |  |  |  |  |  |  |
| 23 | I have had dry eyes  *hatte ich trockene Augen* | .753 |  |  |  |  |
|  |  |  |  |  |  |  |
| 24 | I have had difficulty with bright lights  *hatte ich Schwierigkeiten bei hellem Licht* | .871 |  |  |  |  |
|  |  |  |  |  |  |  |
| 25 | My eyes have been red  *waren meine Augen gerötet* | .823 |  |  |  |  |
|  |  |  |  |  |  |  |
| 26 | I have had pain in/or around the eyes  *hatte ich Schmerzen in den oder um die Augen* | .812 |  |  |  |  |
|  |  |  |  |  |  |  |
| 27 | I have had difficulty reading  *hatte ich Schwierigkeiten beim Lesen* | .827 |  |  | .240 |  |
|  |  |  |  |  |  |  |
| 28 | I have had blurred vision  *habe ich verschwommen gesehen* | .788 |  |  |  |  |
|  |  |  |  |  |  |  |
| 29 | I have been worried about my eyesight  *habe ich mir Sorgen wegen meiner Sehkraft gemacht* | .731 |  | -.259 | .398 |  |
|  |  |  |  |  |  |  |

**Notes:** Pattern matrix of principal component analysis with oblique rotation. N=161. Table only presents factor loadings > 0.2. Clear factor assignments (main factor loading ≥ 0.5 with loading < 0.4 on all other factors) are highlighted in grey colour.
