# Supplementary material for: Translation and psychometric properties of the King’s Sarcoidosis Questionnaire (KSQ) in German language
Source: Health Qual Life Outcomes. 2019 Apr 11;17:62. doi: 10.1186/s12955-019-1131-z (PMC6460543; doi:10.1186/s12955-019-1131-z)
Supplement: Supplementary file 3 — Internal Consistency of the original version (29 items, seven-point Likert scale). (DOCX 35 kb) [file 12955_2019_1131_MOESM3_ESM.docx]

**Supplement table 3. Internal Consistency of the original version (29 items, seven-point Likert scale)**

| **Scale and items** | **N** | **Items** | **Item-Total correlation** | **Mean Inter-Item correlation** | **Cronbach’s alpha** |
| --- | --- | --- | --- | --- | --- |
|  |  |  |  |  |  |
| **General Health Status** | 185 | 10 | 0.44-0.77 | 0.45 | 0.89 |
|  |  |  |  |  |  |
| **Lung** | 188 | 6 | 0.58-0.80 | 0.61 | 0.90 |
|  |  |  |  |  |  |
| **Medication** | 172 | 3 | 0.57-0.76 | 0.62 | 0.83 |
|  |  |  |  |  |  |
| **Skin** | 192 | 3 | 0.69-0.73 | 0.64 | 0.84 |
|  |  |  |  |  |  |
| **Eyes** | 187 | 7 | 0.69-0.80 | 0.60 | 0.91 |
|  |  |  |  |  |  |
