# Supplementary material for: Translation and psychometric properties of the King’s Sarcoidosis Questionnaire (KSQ) in German language
Source: Health Qual Life Outcomes. 2019 Apr 11;17:62. doi: 10.1186/s12955-019-1131-z (PMC6460543; doi:10.1186/s12955-019-1131-z)
Supplement: Supplementary file 4 — Concurrent Validity of the original version (29 items, seven-point Likert scale). (DOCX 35 kb) [file 12955_2019_1131_MOESM4_ESM.docx]

**Supplement table 4.** **Concurrent Validity of the original version (29 items, seven-point Likert scale)**

|  | **Kings Sarcoidosis Questionnaire** | | | | |
| --- | --- | --- | --- | --- | --- |
|  | General Health Status | Lung | Medication | Skin | Eyes |
| **SF-36 Physical Component Score** | .60 | .70 | .27 | .32 | .33 |
| **SF-36 Mental Component Score** | .64 | .27 | .32 | .22** | .18* |
| **Borg scale** | -.53 | -.67 | -.39 | -.30 | -.39 |
| **VAS Dyspnoea** | -.46 | -.71 | -.32 | -.23** | -.27 |

**Notes:** all p<0.001 except ** p<.01 and * p<.05. Interpretation guide: KSQ scales: high values = low burden, SF-36 scales: high values = high manifestation of related dimensions’ content, Borg scale: high values = high dyspnoea, VAS Dyspnoea: high values = high dyspnoea
